# “My Invisalign experience”: content, metrics and comment sentiment analysis of the most popular patient testimonials on YouTube

**DOI:** 10.1186/s40510-017-0201-1

**Published:** 2018-01-22

**Authors:** Christos Livas, Konstantina Delli, Nikolaos Pandis

**Affiliations:** 1 Department of Orthodontics, Academic Centre for Dentistry Amsterdam (ACTA), University of Amsterdam and VU University Amsterdam, Amsterdam, The Netherlands; 2 Department of Oral and Maxillofacial Surgery, University of Groningen, University Medical Center Groningen, Groningen, The Netherlands; 3 Department of Orthodontics and Dentofacial Orthopaedics, School of Dental Medicine, University of Bern, Bern, Switzerland; 4 Private practice, Corfu, Greece

## Abstract

**Background:**

The aim of the study was to investigate the popularity, content of Invisalign patient testimonials on YouTube, as well as the sentiment of the related comments.

**Methods:**

Using the term “Invisalign experience,” the top 100 results on YouTube by view count were screened for English spoken patient videos that attracted comments. Video information (time since video upload, sponsorship), engagement metrics (comments, likes, dislikes, subscriptions), and views were collected. Videos were rated for information completeness (ICS), and comments were classified by origin and content. The emotional loading of the comments was measured using automated sentiment analysis.

**Results:**

The 40 reviewed testimonials scored an average ICS of 3.78 (SD 0.97). ICS, time since upload, and video duration did not appear to significantly influence the number of views, subscriptions, likes, dislikes, and comments. There was a statistically significant difference (*P* = 0.03) between mean positive (2.01, SD 0.95) and negative sentiment scores (− 1.90, SD 1.14). Commenter’s status and overall comment on video were significantly associated with positive sentiment scores. There was a significant association between sponsorship, commenter’s status, overall comment on video, focus of concern, perceived Invisalign’s disadvantages, and increased negative sentiment scores.

**Conclusions:**

Engagement of audience and views of the most popular Invisalign patient testimonials were not significantly influenced by completeness of information, video duration, and lifespan. The sentiment of viewers’ comments about Invisalign treatment was significantly more positive and was significantly associated with their status, content, and sponsorship of videos. Orthodontic trends on YouTube need to be cautiously monitored for planning interventions that improve patients’ knowledge about orthodontics.

## Background

Personal stories of patients in the form of narratives, testimonials, or anecdotes are increasingly becoming available to the public as YouTube videos and discussion posts on social media and peer-support groups. Such anecdotal information is heavily weighed by health consumers when making treatment choices [1]. Health video blogs in video hosting services, also called vlogs, have the potential to impact patients’ psychological health, promote health education for youth and young adults, and improve health information literacy [2].

With over a billion users, YouTube is the fastest growing video sharing Web platform, ranked second in Internet traffic worldwide and in the United States (US) [3]. The educational value of YouTube in dentistry has been reported as underdeveloped and underestimated [4]. The quality and completeness of the dental information on YouTube have been disputed due to the minimal filtering of the uploaded material [5]. Numerous orthodontic videos are also available on YouTube, mostly originating from patients [4], and reflecting a general pro-orthodontics attitude. Referring orthodontic patients to YouTube for relevant audiovisual information resulted in a significant improvement in patient knowledge compared to verbal and written instructions [6].

Given YouTube is being largely used as a primary source of orthodontic information [4], it can be presumed that laypersons interested in contemporary systems like Invisalign are likely to access the video-sharing platform to answer their queries. Nowadays, 89% of US practices perform treatment with clear aligners, while the number of cases has almost doubled within a few years [7]. This upward trend did not slow down regardless of the limited evidence on the effectiveness of Invisalign compared to conventional orthodontics [8]. Interestingly, the manufacturer has recently announced to have reached 4.5 million Invisalign patients worldwide [9]. Without doubt, the growth rate of Invisalign suggests its continued popularity among clinicians and patients. It can be speculated that new communication technologies like YouTube and other social media might have contributed to the broad acceptance by patients, and especially at younger ages.

So far, the available Invisalign information on YouTube videos uploaded by patients-vloggers and the interaction between vloggers and viewers have not been investigated. Therefore, the aims of this study were (i) to determine the completeness of information of the most viewed YouTube patient testimonials regarding Invisalign treatment and the emotional content of viewers’ comments and (ii) to evaluate the association patterns between video metrics, information completeness, and sentiment of comments.

## Methods

### Search strategy

YouTube (http://www.youtube.com) was searched on June 10, 2017, using the phrase “Invisalign experience.” Preliminary YouTube search using the specific search phrase delivered more relevant results than “Invisalign” and “Invisalign testimonials” and therefore selected for the study. Before searching, the computer history and cookies were deleted. No filters regarding video upload date, type, duration, and features were enabled. The initial search returned 61,300 results. To reproduce a standard YouTube search of the average user [10, 11], the first 100 videos sorted by view count were screened for relevance independently by two researchers and any disagreement was resolved in a consensus meeting. The following exclusion criteria were applied: language other than English, videos without comments, no personal experience narrative, irrelevant to Invisalign information, testimonials produced by non-patients, insulting wording, poor audiovisual quality, and duplicates.

### Data collection

Video information (title, hyperlink, vlogger’s name, time since upload), engagement metrics (comments, likes, dislikes, subscriptions), and view metrics were recorded for all eligible videos. The description field of each video was scrutinized for sponsorship statements or endorsement deals.

All videos were viewed and available information regarding clinician’s status, Invisalign cost, treatment procedure, complications and comparison between Invisalign and fixed appliances was extracted. Such information has been previously found to attract attention on social media [12]. Subsequently, two researchers (C.L. and K.D.) measured independently the information completeness score (ICS) of the videos, namely rated the videos with 1 point for each of the five abovementioned topics covered by the vlogger. Commenter’s status was classified according to the categories displayed in Table 1. To prevent misinterpretation of the results regarding the target of the comments, only the ones including the word “Invisalign” were retrieved [13]. Comments were further classified by overall comment, focus of concern, and Invisalign’s disadvantages as perceived by the viewer (Table 2).

**Table 1:** Commenter’s status according to personal experience with or interest in Invisalign

Viewer status
Invisalign patient (former or present)
Fixed appliance patient (former or present)
Experienced both types of appliances
Interested in orthodontics
Getting Invisalign soon
Getting fixed appliances soon
Eager to get Invisalign
Willing to get but cannot afford Invisalign
Regretted getting Invisalign
Other
Not specified

**Table 2:** Comment classification

Overall comment	Focus of concern	Invisalign’s disadvantages
Found video useful/informative	Treatment cost	Pain
Commented on vlogger’s reliability/sponsoring	Complications (pain, lisp, etc.)	Lisp
Positive comment on vlogger’s treatment outcome	Treatment duration/wearing time	Bad odor
Negative comment on vlogger’s treatment outcome	Cleaning aligners/oral hygiene	Wearing time/commitment
Confused Invisalign with retainers	Retention/stability	Dietary consequences
Asked for further information	Efficiency	Public embarrassment
Other	Treatment procedures	Treatment cost
Combination	If Invisalign indicated for own malocclusion/asked for advice	Oral symptoms
No comment	Asked/shared information about own malocclusion/treatment/found similarities	Not indicated for all cases
	Reason for choosing Invisalign	Other (e.g., enamel decalcification)
	Other	Combination
	Combination	No disadvantages mentioned
	No information asked/shared	

### Sentiment analysis

Sentiment analysis or opinion mining is an approach that classifies comments as praise remarks or complaints and can further process these classifications into actionable areas to improve clinical practice [14]. Sentiment analysis of viewers’ comments on Invisalign was carried out using SentiStrength software (version 2.2, copyright Professor M. Thelwall, Faculty of Science and Engineering, University of Wolverhampton, UK), a sentiment analysis tool widely applied to sentiment detection on social network sites [15–19]. SentiStrength [20] is a lexicon-based classifier that synthesizes additional (non-lexical) linguistic information and rules to measure the strength of positive and negative sentiment in short informal English texts on a scale of ± 1 (neutral) to ± 5 (extremely positive/extremely negative). For instance, a text scored with 3, − 5 would signify moderate positive and very strong negative emotions. The full text of comments was imported into the Sentistrength tool, which automatically generated binary reports of positive and negative sentiment strengths. 

### Statistical analysis

Paired *t* test was used to compare the means of positive and negative sentiment scores. Median regression analysis was used to investigate the association between ICS and engagement and view metrics. To determine the association between positive and negative sentiment scores and sponsorship, viewer’s status, overall comment, focus of concern, and disadvantages as perceived by viewers, Pearson’s chi-squared test was used. The significance level was set at 5%. Statistical analysis was performed using a statistical software package (STATA 14.2, Stata Corporation, College Station, Tex, USA).

## Results

**Fig. 1:**
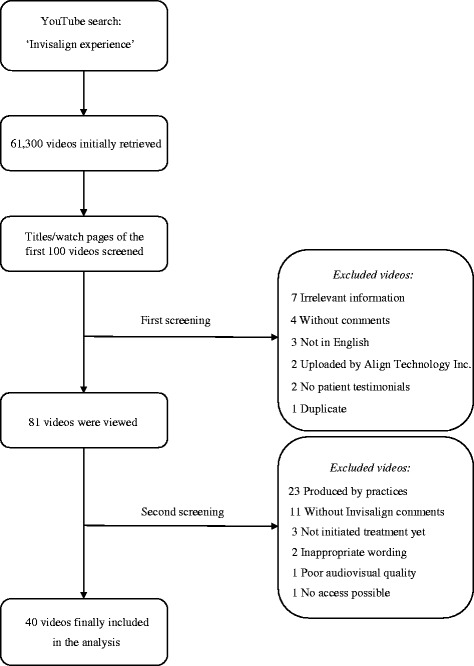
Flowchart diagram of the selection process

YouTube search initially yielded 61,300 results. After screening video titles and watch pages of the first 100 videos, the list of results reduced by 19 videos (Fig. 1). After watching the remainder videos and screening the comments posted by viewers, 40 videos were considered eligible for the study.

### Engagement, view metrics, and sponsorship

The 40 videos were created by 34 unique vloggers with a median of 2605.5 subscriptions. Descriptive statistics (median, interquartile range; IQR) of video duration, lifespan, and engagement metrics are displayed in Table 3. In total, 663 comments were considered for the purposes of the study. Align Technology Inc. was acknowledged in one video, whereas one vlogger revealed to have received discount for treatment costs. Five vloggers disclosed information about the Invisalign provider (name, practice, or Web site). Lack of sponsorship was clearly stated in nine testimonials.

**Table 3:** Descriptive statistics (median, interquartile range; IQR) of video duration, time since upload, and engagement metrics

	Median	IQR
Duration (sec)	526.0	417.5
Time since upload (days)	720.0	937.5
Views	10,940.0	46,529.0
Subscribe	2605.5	7593.5
Likes	94.0	160.5
Dislikes	5.0	25.5
Comments	33.0	56.5

### Presentation, completeness of information, and video scores

The analyzed videos presented a mean ICS of 3.78 (SD 0.97). Eleven videos achieved the theoretical maximum score of 5 points. The results of median regression analysis showed no significant association between ICS, video duration, and time since upload and either engagement metrics or views (Table 4). There was a weak evidence of significant association between video duration and view counts (β-coefficient = 0.16, *P* = 0.10).

**Table 4:** *P* values from the median regression analysis for information completeness score (ICS), views, duration (in seconds), and time since upload (in days)

ICS	*P* value				
	Views	Subscribe	Likes	Dislikes	Comments
3 points	0.99	1.00	0.97	0.96	0.98
4 points	0.98	1.00	0.99	0.99	0.98
5 points	0.90	1.00	0.90	0.79	0.87
Duration	0.85	0.94	0.10	0.51	0.21
Time since upload	0.26	0.87	0.63	0.52	0.67

Half of the vloggers were treated by orthodontists and 17 by dentists. Twenty-nine vloggers were in active treatment at the time the video was recorded. Most vloggers discussed treatment aspects like frequency of changing trays/practice visits, attachments, wearing time, and consultation. Procedures like ClinCheck and Interproximal Enamel Reduction (IER) were scarcely reported. Pain, tooth sensitivity, or discomfort were the most frequent side-effects reported by 22 vloggers. Eleven vloggers complained about oral symptoms, and specifically, mouth dryness and soft tissue irritation. Eleven vloggers had to adjust their dietary habits, while speech was affected (lisp) in ten cases. Fifty percent of the vloggers compared Invisalign and fixed appliances, whereas most of them (14 out of 20) did not have personal experience with braces.

### Comment content and sentiment analysis

Commenter’s status was not specified in 234 cases, while many commenters were related to Invisalign treatment (i.e., 163 former or present patients, 67 motivated to get Invisalign, 41 planning to start treatment soon).

Four hundred sixty-nine viewers did not comment on the video itself, 80 found the video useful/informative, and 31 commented on vlogger’s reliability/sponsoring. Most commenters focused on asking or sharing information about own malocclusion/treatment or even finding similarities with the vlogger’s condition, treatment cost, complications, and efficiency. Twenty-four percent of the viewers shared their view on Invisalign’s disadvantages with most of them mentioning cost, a combination of disadvantages, and pain/oral symptoms.

There was a statistically significant difference (*P* = 0.03) between mean positive (2.01, SD: 0.95) and negative sentiment scores (− 1.90, SD 1.14). There was a significant association between commenter’s status, overall comment on video, and positive sentiment scores (Table 5). Sponsorship, commenter’s status, overall comment on video, focus of concern, and perceived Invisalign’s disadvantages were significantly associated with increased negative sentiment scores (Table 5).

**Table 5:** *P* values of the Pearson’s chi-squared test for sponsorship, commenter’s status, and comment classification

	*P* value	
	Positive sentiment	Negative sentiment
Sponsorship	0.55	< 0.001*
Commenter’s status	< 0.001*	< 0.001*
Overall comment	< 0.001*	0.03
Focus of concern	0.13	< 0.001*
Invisalign’s disadvantages	0.27	< 0.001*

*Statistically significant at *p* < 0.05

## Discussion

This study shows that YouTube users viewed massively videos related to Invisalign treatment and interacted with the creators and fellow viewers. Completeness of information, video duration, and lifespan did not significantly influence the numbers of views, subscriptions, likes, dislikes, and comments. On this basis, the viewing behavior of YouTube audience may be considered unpredictable, determined by random factors.

Almost half of the vloggers were treated by a dentist, a fact that reflects the reality in provision of orthodontic care in the US, where general dental offices have been carrying out up to 48.9% of orthodontic procedures [21]. Hypothetically, a similarly high prevalence of dentists may be expected in the 100,000 certified Invisalign providers around the world [9]. Comparison in the use of Invisalign showed that orthodontists treated generally more Invisalign cases, though dentists were building faster their caseload [22]. General dentists appeared also more willing to treat more complicated malocclusions with Invisalign [22, 23] adhere less to the digital treatment plan, and use fewer auxiliaries, perhaps demonstrating a difference in treatment goals [23].

Treatment procedures as well as complications were among the main themes identified in Internet discussion forums visited by orthognathic patients [24]. Problems experienced with braces were also primarily posted by orthodontic patients in Twitter communication [25]. IER or refinements were rarely described by the vloggers. Nonetheless, the Invisalign process is not relied on aligners alone. It requires the standard use of auxiliaries like attachments, interarch elastics, IER, and altered aligner geometries to improve the predictability of orthodontic movement [8]. Pain and oral symptoms were most frequently reported by vloggers, while they were also highly viewed as Invisalign’s disadvantage by the audience. Notwithstanding, Invisalign tends to cause less pain compared to fixed appliances during the initial stages of treatment, relatively high levels of pain may be anticipated in the first days after insertion [26] or after tray deformation [27]. Several vloggers complained about the need to brush teeth after snacking or tooth sensitivity that made them to adjust the frequency or type of meals (“Invisalign-diet”), which may question the advantage of Invisalign that allows aligner patients “to enjoy all foods” as Align Technology argues [28]. Speech impairment was frequently noted as side effect in the video testimonials. Patients with acrylic plates experienced significantly more pronounced speech difficulties than others with fixed appliances [29] and vacuum-formed retainers [30]. Plenty of viewers appeared to confuse Invisalign aligners with retainers, which might indicate a possible gap in providing sufficient information by specialists during consultation [24]. It is the anonymity of social media that allows patients to communicate topics that they felt uncomfortable to discuss in person or self-perceived inappropriate to ask directly to health care professionals [31].

Narratives are believed to provide essential emotional and social information not usually available through routine resources. Another’s experience helps others to understand their medical condition, cope, and adjust to treatment regimens [32]. Commenters were mainly former, active Invisalign patients or eager to start treatment and shared information about their treatment experience, occlusion, or even found similarities with the vlogger’s description. Like other patients participating in Internet forums [24], Invisalign patients sought online additional information, support, and reassurance from peers undergoing the same process.

A multifaceted audience was actively involved in watching Invisalign testimonials and sharing comments online. Besides channel subscribers or candidates for clear aligners, vloggers, professionals (orthodontists, dentists, practice staff, lab technician), the manufacturer, product promoters (i.e., vibration device claimed to facilitate tooth movement, aligner seating tool), and research recruiters intervened by posting comments. The interest of different viewers’ groups confirms the claimed potential of social media to monitor public response to health issues, identify misinformation, and identify targeted areas for intervention efforts [33].

As YouTube users commonly engage in active discussion by expressing either positive or negative emotions in their messages [34], this study further analyzed the emotional content of the comments about the vlogger’s experience with Invisalign. Overall, the positive loading of the comments was significantly more pronounced than the negative loading, though both positive and negative mean sentiment scores indicated mild emotions. Likewise, a recent Twitter analysis of the patient experience with braces vs Invisalign revealed more positive tweets about orthodontic treatment without significant differences in sentiment between braces and Invisalign tweets [13]. Viewer’s status, comment content, and sponsorship of videos were significantly associated with the sentiments lying behind the comments. Paid patient testimonials are nowadays commonplace across various specialties. Dental, ophthalmologic, and plastic surgery clinics are routinely recruiting patients to help advertise-witness their experience on video testimonials offering treatment discounts [35]. In the present study, the viewers questioned the consumer’s opinion in sponsored reviews and expressed negative comments about the reliability and the motives of the vlogger implying bias driven by financial interests.

Narrative communication through social media can improve users’ learning abilities by providing ideal health role models. On the contrary, sharing information within personal stories may also hide risks, especially when acting as a deterrent for patients from visiting health professionals or the public may not know how to correctly apply online information about their personal health situation. Since videos involving patient experiences are of lower educational value than expert led ones [36], professional associations and academic departments need to take the lead, to develop and disseminate online evidence-based educational videos. Several YouTube and Vimeo video links (i.e., patient testimonials, instructions about eating, brushing during orthodontic treatment, retention, etc.) are available on the Web site of the American Association of Orthodontists (AAO). Additionally, AAO has created its own YouTube channel, which currently hosts more than 50 videos that basically aim to promote the benefits of orthodontics to the larger society. By expanding topics to standard and innovative techniques and materials, users of video sharing platforms will get access to reliable and updated patient education materials. Clinicians should assist patients in navigating social media and embed on the web pages of their practices links to valid information sources [5].

### Study strengths and limitations

This is the first study to investigate information sharing and interaction trends among YouTube users regarding Invisalign treatment. The use of qualitative research in health care enables an in-depth understanding of patients’ thoughts and experiences [24]. Since the researcher is not present during data collection and the conversations analyzed occur naturally, the introduction of bias by the researcher is prevented. Automated sentiment analysis by means of SentiStrength is useful in processing short comments because it extracts both positive and negative sentiments contained in textual statements [37]. Moreover, SentiStrength outperforms other lexical classifiers [36] and therefore may be assumed to have strengthened our methodology.

Like in most studies on consumer health YouTube videos [37], the first five pages of search results were reviewed. A more sophisticated “snowballing” than a sequential screening approach has been recommended instead to identify relevant content through the suggested videos generated by YouTube algorithm [11]. However, it remains still unclear whether such strategy represents the common practice in YouTube searches. Taking into consideration the YouTube dynamics, the search results and the study implications apply only for the specific search date. Albeit the five-point rating scale used in this study was self-developed and not pre-validated, it provided to some degree a measure of the comprehensiveness of information covering treatment aspects that typically concern users of social media and online discussion groups [12, 24, 25]. Future research should further invest in monitoring the orthodontic interests and sentiments of social media users and developing patient education interventions that meet patients’ expectations and needs.

## Conclusions

- There is an intense activity on YouTube on obtaining information regarding treatment with Invisalign aligners.
- The emotional loading of viewers’ comments about Invisalign aligners was significantly more positive and was significantly associated with their status, content, and sponsorship of videos.
- Range of information, video duration, and time since upload did not significantly influence engagement and view metrics, a finding that warns against the unpredictable viewing preferences of YouTube users, and the incomplete information in popular Invisalign patient testimonials.
